# Introduction from the editors

**DOI:** 10.1186/s13223-024-00943-2

**Published:** 2025-01-13

**Authors:** Harold Kim, Anne K. Ellis, Wade Watson

**Affiliations:** 1 Division of Clinical Immunology & Allergy, Department of Medicine, McMaster University, Hamilton, ON Canada; 2 Division of Clinical Immunology and Allergy, Department of Medicine, Western University, London, ON Canada; 3 Division of Allergy & Immunology, Department of Medicine, Queen’s University, Kingston, ON Canada; 4 Division of Allergy, IWK Health Centre, Department of Pediatrics, Dalhousie University, Halifax, NS Canada

In both 2011 and 2018, we published a comprehensive supplement in this journal entitled *A Practical Guide for Allergy and Immunology in Canada*. The primary goal of this supplement was to provide medical students, residents, primary-care physicians, pediatricians, and other healthcare professionals with a comprehensive, yet easy-to-follow, series of peer-reviewed articles covering all of the common conditions we deal with in the field of allergy and immunology. This goal was successfully met, as the articles have been downloaded and accessed hundreds of thousands of times, and hard copies of the supplements were distributed to thousands of medical students and residents across Canada. Given the sustained high readership and interest in these articles over the past 13 years, we believed it was important to publish updates on the topics addressed in the supplement, while also including new articles on food oral immunotherapy (OIT) and secondary immunodeficiency (SID).

The first article in the supplement provides a basic introduction to the main components and function of the immune system and its role in both health and disease, and serves as a background to the allergic and immunopathological disorders discussed in the remainder of this supplement, including asthma, allergic rhinitis, atopic dermatitis (AD), anaphylaxis, food allergy, eosinophilic esophagitis (EoE), urticaria, angioedema, drug allergy, inborn errors of immunity (IEIs) and SID. Asthma remains one of the most common chronic respiratory diseases in Canada [1], and despite advancements in its diagnosis and management, many Canadians with asthma remain poorly controlled [2]. In most patients, however, control can be achieved through the use of avoidance measures and appropriate pharmacological interventions. In recent years, novel biologics and “triple therapies,” which combin an inhaled corticosteroid (ICS), long-acting beta2-agonist (LABA), and long-acting muscarinic antagonist (LAMA) in a single inhaler, have been approved in Canada for the treatment of severe asthma. Allergic rhinitis is another common disorder that is strongly linked to asthma. A “Focused Allergic Rhinitis Practice Parameter for Canada” was published in this journal earlier this year, and we highlight key recommendations and conclusions from this guidance document [3]. AD is one of the most common skin disorders in children [4] and is often the initial step in the “atopic march” (the sequential development of allergic disease manifestations during early childhood), which leads to asthma and/or allergic rhinitis in the majority of afflicted patients [5]. Recently, a new biologic as well as Janus Kinase (JAK) inhibitors have been added to the treatment armamentarium for this condition. EoE is an atopic inflammatory disease of the esophagus that has become increasingly recognized in children and adults, and a biologic has recently been approved in Canada for its treatment. There have also been recent advances in both our understanding and management approaches to urticaria and angioedema and, therefore, separate articles on these disorders have been included in this supplement update.

Drug allergy encompasses a spectrum of immunologically-mediated hypersensitivity reactions that not only affect patient quality of life, but that may also lead to delayed treatment, unnecessary investigations, and even mortality. In this supplement, we provide a background on drug allergy as well as strategies for the diagnosis and management of the most common drug-induced allergic reactions. We also discuss point-of-care tools that have been developed to predict the risk of conducting direct oral challenges in patients with penicillin or sulfonamide antibiotic allergy.

Anaphylaxis is an acute, potentially fatal systemic hypersensitivity reaction with varied mechanisms and clinical presentations. Although prompt recognition and treatment of anaphylaxis are imperative, both patients and healthcare professionals often fail to recognize and diagnose its early signs. We provide an update on the causes, modulating factors and clinical features of anaphylaxis as well as strategies for the accurate diagnosis and management of the condition. Immunoglobulin E (IgE)-mediated food allergy is a leading cause of anaphylaxis-related emergency department visits and is the most common cause of anaphylaxis in children [6–10]. Accurate diagnosis and appropriate management of IgE-mediated food allergy are critical since, in some patients, accidental exposure to minute quantities of the culprit food may result in anaphylaxis. In this supplement, we provide an updated review on the pathophysiology, diagnosis, and management of IgE-mediated food allergy. The non-IgE-mediated food allergies that result in gastrointestinal (GI) symptoms include food protein–induced allergic proctocolitis (FPIAP), food protein–induced enteropathy (FPE), and food protein–induced enterocolitis syndrome (FPIES). Typically, these non-IgE-mediated food allergies begin in infancy or early childhood, although FPIES can present across the lifespan, with increasing reports in adults in recent years [11–16]. In this supplement, we outline the clinical manifestations, epidemiology, pathophysiology, diagnosis, management, and prognosis of these non-IgE-mediated food allergies.

Delayed introduction of allergenic foods to infants, followed by years of hesitancy to introduce these foods early, has likely contributed to the increase in food allergy prevalence in recent decades. Given the high burden associated with food allergy, primary prevention has become an important public health goal. In this supplement, we review current evidence and guideline recommendations that emphasize the importance of both early introduction and regular consumption of common allergens for the primary prevention of food allergy. Food OIT is a management option for failed primary prevention in infants and is also an option for the treatment of IgE‐mediated food allergy. It involves administering gradually increasing doses of an allergenic food over time (under medical supervision) with the goal of desensitizing an individual to the food allergen. In this supplement, we provide an overview of this form of therapy and discuss the role that non-allergist clinicians can play in caring for patients undergoing OIT. Allergen immunotherapy (AIT) is the only potentially disease-modifying therapy for allergic disease not related to foods, and has been proven to be effective for the treatment of allergic rhinitis/conjunctivitis, allergic asthma and stinging insect hypersensitivity. We provide an update on the indications and contraindications, patient selection criteria, and the administration, safety and efficacy of AIT.

This supplement also provides an updated review of primary immunodeficiencies, now often referred to as inborn errors of immunity (IEI), which represent a large heterogeneous group of disorders that result from deficiencies in immune system development and/or function. Since many IEIs present as “routine” infections, they may initially go undetected in the primary-care setting. Accurate and timely diagnosis of these disorders requires a high index of suspicion and specialized testing, and consultation with a clinical immunologist is imperative for preventing significant disease-associated morbidity and mortality and improving outcomes in patients with these conditions. SID arise from factors unrelated to IEI. There are numerous causes of SID, which can impact both the innate and adaptive immune responses and/or multiple components of the immune system simultaneously. In this supplement update, we provide a broad overview of the various conditions and treatments that can cause SID, as well as the evaluation and management of patients with these immunodeficiencies.

For each of the above-mentioned articles, key take-home messages are summarized for quick reference and, where applicable, easy-to-follow flow charts, tables and algorithms are provided to assist clinicians in the identification, diagnosis and treatment of these common allergic diseases. We are confident that readers will not only find this updated supplement educational and informative, but that it will also provide clinicians with a solid base of knowledge and skills in allergy and immunology which they can then incorporate into their respective clinical practices to help improve the care and management of patients with allergic disease. It is also our hope that this supplement will spark further interest in these conditions and in our specialty.

We thank all of the authors who set aside time from their numerous commitments to write and review these informative articles, the peer reviewers for providing their highly-valued feedback, and the Canadian Society of Asthma and Clinical Immunology (CSACI) and other sponsors who provided the support needed for the development of this important educational initiative. Finally, in tribute to our dear friend and esteemed colleague, Dr. Richard J. Warrington, we dedicate this supplement to his memory. Dr. Warrington was the driving force behind the development and publication of the first two editions of *A Practical Guide for Allergy and Immunology in Canada*, and his vision and leadership have left an enduring legacy in the field of allergy and immunology (please see dedication to Dr. Warrington in this supplement).

We genuinely hope you find this updated supplement enjoyable and valuable.

Harold Kim, MD, FRCPC.

Anne K. Ellis, MD, MSc, FRCP(C), FAAAA.

Wade Watson, MD, MEd, FRCPC.

## Data Availability

Not applicable.

## Abbreviations

AD: Atopic dermatitis
AIT: Allergen immunotherapy
CSACI: Canadian Society of Asthma and Clinical Immunology
EoE: Eosinophilic esophagitis
FPE: Food protein–induced enteropathy
FPIAP: Food protein–induced allergic proctocolitis
FPIES: Food protein–induced enterocolitis syndrome
GI: Gastrointestinal
IEI: Inborn errors of immunity
IgE: Immunoglobulin E
ASA: Acetylsalicylic acid
NSAIDs: Non-steroidal anti-inflammatory drugs
OIT: Oral immunotherapy
SID: Secondary immunodeficiency
